# When and How May We Destroy the Dental Pulp?

**Published:** 1901-06-15

**Authors:** Nelville S. Hoff


### WHEN AND HOW MAY WE DESTROY THE DENTAL PULP?

BY NELVILLE S. HOFF, D.D.S.

Read before the Detroit Dental Society May 6, 1901.

In propounding this question I do not presume to be able to bring to you any new or great enlightenment on this somewhat trite subject. My excuse for bringing up the subject at all is the fact that there is a great diversity of opinion and practice about this operation. Since the advent of crown and bridge work into dental practice it has become a matter that it seems to me should be more thoroughly considered. If it is practiced, some definite rules for guidance should be adopted. While I realize the utter impracticability of coming to any one single practice, I entertain the hope that this question may not down until *some* clear guiding principles shall have been established, whereby we may have a basis of judgment in coming to the proper conclusion as to what and how to do in any ordinary case. I hope that I may so present this matter to you as to bring out in discussion your practices and the reasons which sustain you in them. You will therefore, I trust, pardon me, should I seem to present the subject in a somewhat pedantic manner. I should very much prefer to deal with it from the clinical standpoint, but by doing so the object of my own thought would not be secured. I shall, however, expect that you will be kind enough to supply this omission in the discussion which follows.

In order that I may bring the subject to your attention in a somewhat systematic way and for a more logical presentation I will try to present the first part of the query first under the classification of "When?"

As a first proposition I may say that we should destroy all living pulps that can not be kept in a normal condition in teeth requiring filling, or which may be needed as supports to artificial substitutes.

There is nothing probably more distressing to a patient than an artificially protected and irritable pulp. It is ready at every let down of the general system, from sickness, fatigue, or mental excitement or worry, to "bob up serenely." And as it can express its egotism in a more persistent and attractive way than most other sensible organs, it gets first notice. It is like the ubiquitous telephone bell, it must be answered, no matter what other pressing matters are on hand, and consequently it gets itself generally disliked for its excessive and imperative demands. Next to badly fitting plates we get more unjust criticism and probably lose more prospective business from teeth that ache from irritable pulps under crowns or fillings, than from lack of thorough work or acute business methods, such as heavy fees and prompt collection of bills. What are some of these cases, or how can we diagnose these conditions so that we may avoid them? It is difficult to always decide this question on the instant, there are many factors that will need to be considered. Sometimes we reach our conclusion almost intuitively, or because we are familiar with our patient's idiosyncrasies or those of other members of his family. We may come to judgment by a somewhat systematic process of elimination; reasoning not only from former experiences under similar conditions with other patients, or with the same patient in other operations. For instance, we find that in certain varieties of decay there is no well-defined limit to the extent of tooth destruction, the whole tooth seems badly disorganized, and while the pulp may not be really uncovered, the disintegration of the tooth structure is such as enables us to predict the probable effect of placing a filling in the cavity that will not irritate, or a crown that will not necessarily produce uncomfortable sensations, to say nothing of the almost certain probability of being unable to sterilize such a tooth, and thus prevent further decay.

We sometimes have small exposures or near approach to the pulp in teeth of persons who seem to have no considerable vital resistance or recuperative force; wounds heal slowly and with considerable sloughing. This condition makes a very unfavorable prognosis and gives ample reason for radical measures, even though temporary or inexpensive fillings are to be inserted.

We have another class of teeth that are liable to give us undesirable results: Exposures or near approach in densely calcified teeth where the decay line is sharply marked, but the patient has a general nervous, or worse, a nervo-bilious temperament; exceedingly sensitive and always irritable. These pulps when capped die, but not quickly or easily. Mechanical or thermal changes always produce irritation which leads to inflammation and ultimately to the death of the pulp—or the dentist, if the patient does not go first. The sanguinary temperament sometimes produces undesirable results, but usually these patients offer favorable promise, because the vigorous circulation keeps up active nutrition in the pulp and aids sterilization. When the pulp of a sanguinary person dies it goes quickly, but makes a considerable disturbance in doing so.

The above cases illustrate some of the most difficult classes of cases we have to deal with. There are many other cases where there is a considerable exposure and an evident infection and already a considerable loss of pulp tissue by suppuration, or the pulp is congested and inflamed, or its tissues have changed to an abnormal character. Most of us know how difficult, under the most favorable conditions, it is to preserve the vitality of such pulp. Fifteen years ago this was the battle-ground, and we even ventured to amputate portions of such pulps, and endeavored to and thought we did sometimes succeed in restoring them to health and normal functions. But the seat of war has changed now to the upper heights, and we hesitate to make attempts to preserve pulps that have only slight exposures, in teeth having an unfavorable history or environment. All this change has come about not so much because of the fact that these early efforts were entirely fruitless, but because of the fact that physical and even climatic conditions brought so many failures that many dentists, because of skillful manipulative ability or therapeutic resources, became discouraged and then anarchistic in their practices and proclamations. But I am confident that the most important reason for the recent avalanche of public sentiment in the direction ot the radical destruction of the pulps, comes with the advent of crown and bridge work and the use of antiseptic remedies, as well as the influence of the local anesthetics, which make it easy to accomplish this desirable end. There is ample justification for deciding doubtful cases in favor of pulp extermination when crowns and bridges are to be inserted, particularly where several teeth are involved in a bridge, and especially if the appliance has one or more attachments by means of post crowns. The difficulty of removing such pieces of work when once set, and the necessary destruction of an expensive and efficient piece of work is so great, as to make such a proceeding very unfortunate and undesirable. It is not often possible to make a useful and esthetic bridge, in such a way that it can be readily removed, or that easy access may be had to a pulp through the crown. This fact is so manifest to the bridge workers of any considerable experience that they make it almost a rule to destroy pulps in all teeth from which they are compelled to remove the enamel and have a near approach to the pulp. It seems to me that owing to the possibility of painless and ready removal of pulps, and of the excellent means for thoroughly sterilizing and filling the roots, skillful operators of the present time are justified in removing all pulps from teeth where there is any strong probability that they will not remain in a normal condition under the artificial environment to which they are to be subjected.

How shall we destroy or remove the condemned?

Poison, electrocution and amputation are the accepted ideas and practice. Destructive cauterants have and still hold a prominent place as agents for devitalizing pulps. The mineral acids, carbolic acid and arseneous acid, each have advocates and uses.

The mineral acids are seldom used, except where prompt action is desired, or where it is not possible to confine the slower acting cauterants to the pulp for a sufficient length of time to secure the desired result. In incisor teeth abraded on the cutting edges, where the pulp has been exposed by chemical and mechanical wasting of the tooth substance, leaving no cavity for the confinement of the remedy for a prolonged period, nitric and sulphuric acid are used, with instrumentation to extirpate pulps of teeth worn down to the gum line. The great and serious objection to the use of these agents is the excessive pain they produce. They are, however, seldom used, and only in emergency cases.

Carbolic acid is a more efficient cauterant, and is not painful. The most serious objection to its use is the fact that it is readily decomposed by organic tissues to insoluble compounds and must be frequently applied, as it acts slowly. It is not absorbed easily or deeply, and consequently acts superficially. It is liable to saturate the tooth with a comparatively insoluble pigment which discolors the tooth. It, however, keeps the tooth sterile, thus preventing septic secondary results.

Arsenious acid is a more effective and reliable cauterant for pulp destruction than either of those mentioned. It is a very destructive and somewhat irritant cauterant. Its narcotic effects are inferior to carbolic acid. It is only slightly soluble in the fluids of the pulp, but it is decomposed by the pulp tissue into a soluble arsenical compound which is easily absorbed, so that one application will usually destroy a pulp completely. Should excessive quantities be used, or it be kept in the tooth too long, it will penetrate the apical foramen and destroy the tissues surrounding the root of the tooth. It is particularly liable to do this when used in the teeth of very young persons, because of the large apical openings. Its application is painful, but this tendency may be overcome by combining narcotizing drugs with it. With most practitioners it is the one remedy upon which they rely. There are objections to its use which ought to cause its abandonment in all but exceptional cases. It is in most cases very painful, and it causes a soreness of the dentin which requires several days to overcome by palliative treatment. There is a possibility of its being washed out of the tooth cavity during mastication, causing poisoning of the gum tissues, which it is exceedingly difficult to restore to normal condition. It may set up an irritation of the peridental membrane, because of the extravasation of the arsenic through the apical foramina, or the dentinal tubuli, which sets up an uncomfortable, nagging sort of toothache that will not pass to suppuration, nor will it respond favorably to any local anodyne treatment; the patient must "grin and bear it" until nature establishes a more normal condition. While it acts definitely and favorably in most cases, the objections enumerated above will indicate some more favorable agent when it shall have been found.

*Electrocution.*—This has again come into very great favor. Some forty years ago, extracting of tooth pulps and treatment of alveolar inflammatory conditions was practiced with some success by the application of electricity, at the suggestion of Dr. Richardson, of England. The process of electric osmosis was not as clearly understood as it is to-day, and yet we find Dr. Richardson recommended using aconite, alcohol and other substances to assist the galvanic current in obtunding sensitiveness of the gums in extraction. The method of using the electric current was, however, on the principle that it had a sedative effect on sensitive tissues, due to the electrolytic action of the fluid contents of the tissues. There is probably no question but that electrolosis does so change the existing tissues as to modify normal functions, and, if excessively or injudiciously used, will destroy the vitality and produce sloughing of the soft tissues. It was this fact, together with the fact that no proper appliances were at command with which the electricity might be employed under definite conditions, which caused its abandonment by the dental profession, for many years, or until the recent age of modern electricity and the discovery of a suitable electrolyte for local anesthesia in cocain. The principle of cataphoresis has developed wonderful possibilities in the future treatment of dental diseases. It so far has had no more effective employment than as an anesthetic in obtunding dentin and removing pulps from teeth without pain. With it pulps may be removed from teeth safely, painlessly and expeditiously. The operation of destroying a pulp and filling the root canal can now be well done in a half hour, where it formerly required several sittings and an expense of several days' time. This method of electrocution is perhaps one of the best for general practice that we have at our command. It is safe, and no secondary effects are likely to interfere with the complete success of operations made in this way. Electricity is sometimes used to destroy pulps in the way of destructive cauterization; a heated cautery can be so manipulated that where easy access and roots of considerable size are to be treated the cautery may be used to good advantage. The difficulties are that the cautery can not be used in teeth of difficult access, and in small or tortuous root canals, or in the contracting portions of such roots as the lower incisor, or the anterior root of the first lower molar. It is not painless, although the pain is not so intense as from actual cautery expedients.

Under *Amputation* there are several ideas, some of which are of sufficient importance to demand notice. One of these is the well-known and successful practice of driving into a pulp canal a wedge of wood of the shape and size of the pulp canal which contains the pulp to be removed. The peg or wedge is not useful, however, except in the case of teeth having straight and single roots and where direct and easy access may be had. The method is to drive the peg suddenly and with force by a sharp mallet blow into the canal, displacing the pulp. The method is said to be painless, and may be in some cases. The cases where it can be made practicable are so few that it has been abandoned for more universal methods which accomplish the same practicable result and without the intense nervous shock which is liable to take place from a bungling operation, or when a carefully planned and executed purpose is thwarted by some unforseen condition which modifies the whole procedure. The amputation of pulps under the influence of a general or local anesthetic, is one of the best and most successful methods of the present time.

Pulps may be removed while the patient is under the influence of a general anesthetic, such as nitrous oxid, or ether, or brom. ether. This method of removing pulps has many things to be said in its favor. The use of local obtundants or anesthetics is a more recent and practical idea. All kinds of local obtundants have been used for this purpose, freezing with chlorid of ethyl, chlorid of methyl, ether spray, etc., have each been used successfully. But none of these local obtundants have been so accredited as cocain. As a local anesthetic it is without a peer. Cocain may be usefully employed for this purpose in several ways. It may be applied in crystal form to the pulp until it has by absorption so anesthetized the pulp that it can be operated upon without pain. It may be used with a cataphoric instrument, in which capacity it probably acts as a local anesthetic or obtundant and also as an electrolyte. Probably the most acceptable method of using it is by injecting it directly into the pulp or the gum and extracting the pulp while the anesthesia persists, from either the "PRESSURE METHOD" or hypodermic injection. Used in this way cocain is harmless, efficient and probably the quickest method of anesthetizing live pulps. Harmless because no toxic effects will result, even from large doses of concentrated solution injected directed into the pulp of a tooth. I have found that 1 or 2 percent solutions are quite sufficient for ordinary work. There can be no danger from its use in so dilute a solution.

Eucain is a close rival of cocain, and bids fair to share the honors with cocain in pulp work. It creates more irritation than cocain, consequently it can not be used in soft tissues that are to be resuscitated as it is liable to produce cellulitis. For pulp work it can be used in as high as 10 percent solution, although 5 percent would answer as well. I have not had satisfactory results in using the other local anesthetics by this method, although they are said to be effective when so used. None of them have equal powers with cocain, and there is no reason why they should supersede it, except that they may be less toxic. This is a possibility which, however, has so little bearing, when cocain is intelligently used, that it is hardly worth while discussing.

### DISCUSSION.

Dr. Collins: All pulps should be destroyed when they have lost their usefulness. In small exposures I generally advise removal. When nearly exposed, I generally remove if much pain had been previously experienced. When bridge work of any extent is to be inserted, the removal of pulps is essential, but not so much advised in the case of a single gold crown.

Referring to "how we may destroy pulps," I consider two methods sufficient to classify, namely, poisons and anesthetics. In using poisons, arsenic and carbolic acid in combination with sulphate of morphia, is very good for adults.

For children carbolic acid is sufficient. Never had practiced amputation, but think well of the pressure method.

Dr. Noble: I use pressure anesthesia with cocain, and think also hypodermic injection of the roots a very desirable method in cases where direct access is practicable.

Dr. Oakman: The use of cocain in many cases is invaluable and the most expeditious of all methods of pulp extirpation. I prefer arsenic mixed with carbolic acid for all superior molars and inaccessible cavities.

Dr. Young: I always uncover pulps that have ached. I frequently find pus present in such cases.

In uncovering pulps I first apply vapo-cocain with pressure, and when the pulps are thoroughly exposed, if removal is necessary, I use pressure anesthesia. Would not advise arsenic to be left in a tooth longer than twenty-four hours.

